# Playstation Thumb: Frictional Dermatitis Caused by Excessive Video Game Playing

**DOI:** 10.5826/dpc.1004a92

**Published:** 2020-10-26

**Authors:** Jaime Piquero-Casals, Daniel Morgado-Carrasco, Juan Francisco Mir-Bonafé, Eduardo Rozas-Muñoz

**Affiliations:** 1 Department of Dermatology, Dermik, Clínica Dermatológica Multidisciplinar, Barcelona, Spain; 2 Department of Dermatology, Hospital Clínic de Barcelona, Universitat de Barcelona, Spain; 3 Department of Dermatology, Hospital Son Llàtzer, Palma de Mallorca, Spain; 4 Department of Dermatology, Hospital de San Pablo, Coquimbo, Chile

**Keywords:** playstation thumb, texting thumb, video game, nintendinitis, frictional dermatitis, tech thumb

## Case Presentation

**Figure 1:**
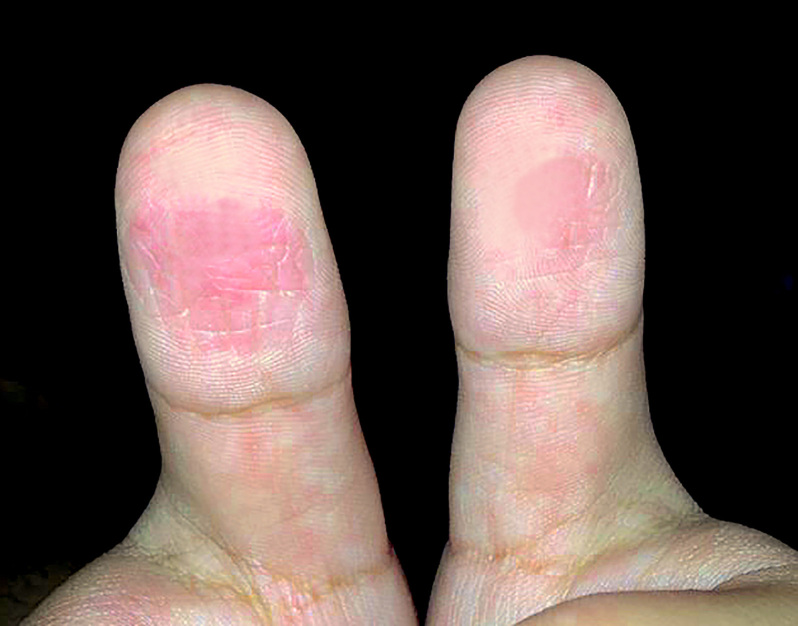
Erythema, lichenification and fissures on the tips of both thumbs.

An otherwise healthy 9-year-old boy presented with itching and painful lesions on both thumbs of 5 months’ evolution. He had received treatment with high-potency topical corticosteroids with partial improvement. Physical examination showed erythema, lichenification and fissures on the tips of both thumbs (Figure 1). Patch testing was performed, with negative results. After exhaustive questioning, the parents stated that the child usually spent several hours per day playing video games. The boy also admitted to being “addicted to PlayStation games.” A diagnosis of frictional dermatitis was made. The lesions completely resolved following 2 weeks of forced abstinence from video gaming.

## Teaching Point

Among the cutaneous manifestations of excessive video gaming, video gamer thumb or “playstation thumb” refers to the presence of hyperkeratosis, blisters, and petechiae on the tips of the thumbs [1]. Multiple cutaneous disorders can arise from excessive use of electronic devices [2], and a high index of suspicion is necessary to allow an early diagnosis.

## References

[1] Wolf R, Wolf D (2014). Playstation thumb. Int J Dermatol.

[2] Jalink MB, Heineman E, Pierie JP, ten Cate Hoedemaker HO (2014). Nintendo related injuries and other problems: review. BMJ.

